# One Episode of Self-Resolving *Plasmodium yoelii* Infection Transiently Exacerbates Chronic *Mycobacterium tuberculosis* Infection

**DOI:** 10.3389/fmicb.2016.00152

**Published:** 2016-02-15

**Authors:** Jannike Blank, Lars Eggers, Jochen Behrends, Thomas Jacobs, Bianca E. Schneider

**Affiliations:** ^1^Division of Coinfection, Priority Research Area Infections, Research Center BorstelBorstel, Germany; ^2^Fluorescence Cytometry Core Facility, Research Center BorstelBorstel, Germany; ^3^Department of Immunology, Bernhard Nocht Institute for Tropical MedicineHamburg, Germany

**Keywords:** *Mycobacterium tuberculosis*, *Plasmodium yoelii* malaria, tuberculosis, co-infection, mouse model

## Abstract

Malaria and tuberculosis (Tb) are two of the main causes of death from infectious diseases globally. The pathogenic agents, *Plasmodium* parasites and *Mycobacterium tuberculosis*, are co-endemic in many regions in the world, however, compared to other co-infections like HIV/Tb or helminth/Tb, malaria/Tb has been given less attention both in clinical and immunological studies. Due to the lack of sufficient human data, the impact of malaria on Tb and *vice versa* is difficult to estimate but co-infections are likely to occur very frequently. Due to its immunomodulatory properties malaria might be an underestimated risk factor for latent or active Tb patients particularly in high-endemic malaria settings were people experience reinfections very frequently. In the present study, we used the non-lethal strain of *Plasmodium yoelii* to investigate, how one episode of self-resolving malaria impact on a chronic *M. tuberculosis* infection. *P. yoelii* co-infection resulted in exacerbation of Tb disease as demonstrated by increased pathology and cellular infiltration of the lungs which coincided with elevated levels of pro- and anti-inflammatory mediators. T cell responses were not impaired in co-infected mice but enhanced and likely contributed to increased cytokine production. We found a slight but statistically significant increase in *M. tuberculosis* burden in co-infected animals and increased lung CFU was positively correlated with elevated levels of TNFα but not IL-10. Infection with *P. yoelii* induced the recruitment of a CD11c^+^ population into lungs and spleens of *M. tuberculosis* infected mice. CD11c^+^ cells isolated from *P. yoelii* infected spleens promoted survival and growth of *M. tuberculosis in vitro*. 170 days after *P. yoelii* infection changes in immunopathology and cellular immune responses were no longer apparent while *M. tuberculosis* numbers were still slightly higher in lungs, but not in spleens of co-infected mice. In conclusion, one episode of *P. yoelii* co-infection transiently exacerbated disease severity but had no long-term consequences on disease progression and survival of *M. tuberculosis* infected mice.

## Introduction

Tuberculosis (Tb) and malaria are the most prevalent bacterial and parasitic infections in humans, respectively, and continue to be major causes of morbidity and mortality in impoverished regions in the tropics. The causative agent of Tb, *Mycobacterium tuberculosis* is carried by an estimated 2–3 billion people globally, but in most cases it lies dormant and the immune system is able to prevent it from spreading in the body (WHO, 2015). A relatively small proportion (5–15%) of *M. tuberculosis*-infected people will develop active disease during their lifetime. However, the immune system fails to achieve sterile eradication of the tubercle bacillus. The enormous reservoir of latent Tb patients constantly leads to new active Tb cases and transmission of the disease, thus perpetuating the epidemic. The reasons why some people develop active Tb, while others contain the infection remain enigmatic. Reactivation can occur after years or decades of clinical latency, and the risk of reactivation increases with conditions that modulate the immune status of the host such as disease (most prominent HIV/AIDS), drug treatment, age, or malnutrition (O’Garra et al., 2013).

Malaria is highly prevalent in populations where *M. tuberculosis* is endemic. 3.3 billion people are at risk of being infected with the causative agent, protozoan parasites of the genus *Plasmodium*. In 2013, approximately 200 million cases of malaria led to 600,000 deaths, predominately in young children under the age of 5 and pregnant women in sub-Saharan Africa (Murray et al., 2012). In malaria-endemic areas, immunity slowly develops over time, which does not prevent reinfection but limits parasite density and symptoms. Consequently, the majority of *Plasmodium* infections in adults are mild or asymptomatic (Bousema et al., 2014). However, the public health impact of malaria goes beyond the direct burden of the disease. Both symptomatic and asymptomatic malarial infections can cause immune modulation, which has long been discussed to account for constant malaria reinfections, reduced vaccine efficacy as well as for an increased susceptibility to secondary infections (including bacteria such as *Salmonella* or viruses such as Herpes virus and Epstein-Barr virus; Greenwood et al., 1972; Bomford and Wedderburn, 1973; Warren and Weidanz, 1976; Williamson and Greenwood, 1978; Correa et al., 1980; Brasseur et al., 1983; Whittle et al., 1984; Cook, 1985; Mabey et al., 1987; Hviid et al., 1991; Cunnington and Riley, 2010; Walther et al., 2012). Epidemiological studies showed that death rates in adults and children declined considerably when the incidence of malaria was reduced, while the entire reduction in death rates could not be directly attributed to malaria (Enwere et al., 1999; Kleinschmidt et al., 2009; Cunnington and Riley, 2010). This was already noted back in the 19th century, where post-mortem examinations revealed that deaths secondary to malaria were at least as great as mortality directly attributed to malaria infection and correlated with co-endemic infectious diseases such as Tb, pneumonia and diarrhea (Shanks et al., 2008). In line with this is a more recent clinical study in Guinea-Bissau, which reported improved clinical outcome and reduced mortality among severely ill Tb patients after malaria prevention had been carried out (Colombatti et al., 2011).

Most of the experimental studies on co-infection between mycobacteria and *Plasmodium* focus on the unspecific protective effects of mycobacterial infections against malaria (Clark et al., 1976; Murphy, 1981; Matsumoto et al., 2000; Page et al., 2005; Mueller et al., 2012). The majority of such studies addressed the question as to whether the widely used Tb vaccine strain *M. bovis* Bacille Calmette Guerin (BCG) confers non-specific protection against subsequent *Plasmodium* infection (Clark et al., 1976; Smrkovski and Strickland, 1978; Matsumoto et al., 2000; Leisewitz et al., 2008; Parra et al., 2013) since BCG has been associated with reduced child mortality from causes other than Tb (Roth et al., 2005, 2006a,b; Shann 2010, 2011). In contrast, only two experimental studies including our own investigated the outcome of virulent *M. tuberculosis* infection in the context of malaria co-infection in the mouse model and indeed found the control of *M. tuberculosis* to be impaired in the presence of different rodent malaria parasites (Scott et al., 2004; Mueller et al., 2012). In our previous study, we reported that co-infection with *P. berghei* NK65, a lethal strain causing malaria-associated acute respiratory distress syndrome in C57BL/6 mice (Van den Steen et al., 2010), was associated with enhanced inflammatory immune responses and tissue pathology, hypercytokinemia and altered T-cell responses which resulted in impaired control of chronic *M. tuberculosis* infection (Mueller et al., 2012). Similarly, co-infection with the non-lethal strain of *P. yoelii* interfered with the containment of *M. tuberculosis* although to a lesser extent (Scott et al., 2004). The immunological mechanisms have not been studied in detail.

## Materials and Methods

### Ethics Statement

Animal experiments were approved by the Ethics Committee for Animal Experiments of the Ministry for Agriculture, Environment, and Rural Areas of the State of Schleswig-Holstein (Kommission für Tierversuche/Ethik-Kommission des Landes Schleswig-Holstein) under the license 33–3/10 (“Die Auswirkung von Tuberkulose auf die Pathogenese und Immunantwort bei Malaria im Rahmen einer Koinfektion in der Maus”/“The impact of tuberculosis on pathogenesis and immune responses to malaria in an experimental co-infection mouse model”).

### Mice, Bacterial Infection, and Colony Forming Units

For all *in vivo* experiments female C57BL/6 mice aged between 6 and 8 weeks were used, which were obtained from Charles River Laboratories. Mice were maintained under specific barrier conditions in BSL 3 facilities. For all *in vitro* experiments female and male C57BL/6 wild-type and female transgenic OT2 mice aged between 8 and 20 weeks were used, bred in the animal facility of the Research Center Borstel.

*Mycobacterium tuberculosis* H37Rv was grown in Middlebrook 7H9 broth (BD Biosciences) supplemented with 10% v/v OADC (Oleic acid, Albumin, Dextrose, Catalase) enrichment medium (BD Biosciences). Bacterial cultures were harvested, resuspended in PBS/10% glycerol, and aliquots were frozen at –80°C until later use. Viable cell counts in thawed aliquots were determined by plating serial dilutions onto Middlebrook 7H11 agar plates supplemented with 10% v/v heat-inactivated bovine serum, 0.1% w/v asparagine and 0.5% v/v glycerol followed by incubation at 37°C.

For infection of experimental animals, *M. tuberculosis* stocks were diluted in sterile distilled water at a concentration providing an uptake of 200 viable bacilli per lung. Infection was performed via the respiratory route by using an aerosol chamber (Glas-Col, Terre-Haute, IN, USA). Animals were exposed for 40 min to an aerosol generated by nebulizing the prepared *M. tuberculosis* suspension. The inoculum size was quantified 24 h after infection by determining bacterial loads in the lungs of infected mice. Bacterial loads in lung, liver, and spleen were evaluated at different time points after aerosol infection by mechanical disruption of the organs in 0.05% v/v Tween 20 in PBS containing a proteinase inhibitor cocktail (Roche) prepared according to the manufacturer’s instructions. Tenfold serial dilutions of organ homogenates in sterile water/1% v/v Tween 80/1% w/v albumin were plated onto Middlebrook 7H11 agar plates and incubated at 37°C. Colonies were enumerated after 3–4 weeks.

### Parasitic Infection

*Plasmodium yoelii* 17NL (non-lethal) was maintained by regular passage in NMRI mice. For cryopreservation, blood was collected from highly parasitemic mice, and aliquots were stored in liquid nitrogen in a solution of 0.9% NaCl, 4.6% sorbitol, and 35% glycerol. Experimental naïve mice or animals pre-infected for 30 days with *M. tuberculosis* were infected intraperitoneally (i.p.) with 1 × 10^5^ *P. yoelii*-infected RBCs obtained from a homologous donor, which had been infected from frozen stock. Parasitemia was determined on Giemsa-stained blood smears from tail blood every 2 to 3 days. Moreover, mice were checked for *P. yoelii*-induced anemia based on the hemoglobin concentration in the blood. For this, tail vein blood was collected and diluted in Drabkin’s Solution supplemented with Brij L23 Solution (Sigma–Aldrich). Optical density of hemoglobin was measured at 540 nm.

### Cell Isolation and Purification from Lungs and Spleens

Mice were sacrificed 51 or 200 days p.i. with *M. tuberculosis* and perfused intracardially with 20 ml PBS to remove circulating leukocytes from the tissue. Lungs were digested in 100 μg/ml DNase I (Roche) and 50 μg/ml Liberase TL (Roche) in RPMI for 90 min and passed through a 100 μm pore size cell strainer to obtain a single cell suspension. Spleens were passed through a 100 μm pore size cell strainer. Remaining erythrocytes in lung and spleen cell suspensions were lysed (155 mM NH_4_Cl, 10 mM KHCO_3_, 0.1 mM EDTA in H_2_O) and cells were resuspended in RPMI 1640 supplemented with 2 mM L-glutamine, 1% v/v Hepes, 50 μM β-mercaptoethanol and 10% v/v heat-inactivated fetal calf serum (complete RPMI 1640 medium). Cell numbers were determined with the Vi-CELL Cell Viability Analyzer (Beckman Coulter).

### Flow Cytometry

For flow cytometric analysis of surface markers and intracellular cytokines, single cell suspensions of lungs, spleens, or cell cultures were stained with optimal concentrations of the following specific antibodies: CD45-V450, CD4-V500, CD8-V450, and CD62L-APC from BD Biosciences, CD3-PerCP-Cy5.5, CD4-BV510, CD4-PE-Cy7, CD8a-FITC, CD44-FITC, CD19-PE, CD80-AF488, CD86-APC, Ly6G-APC-Cy7, CD11c-PE-Cy7, NK1.1-PE-Cy7, TNFα-Pacific Blue, IFNγ-PerCP-Cy5.5, IL-17A-PerCP-Cy5.5, IL-2-PE-Cy7, and IL-10-PE from BioLegend and CD90.2-eFluor780 from eBioscience. Data were acquired on a FacsCantoII^®^ flow cytometer (BD Biosciences) equipped with a 405, 488, and 633 nm laser and analyzed with the FCS Express software (DeNovo™ Software).

### Intracellular Cytokine Staining

Single cell suspensions of lungs or spleens (1 × 10^6^) were stimulated 4.5 h with αCD3e/αCD28 (BioLegend; 5 μg/ml, respectively) in the presence of GolgiPlug™ (BD Biosciences, contains Brefeldin A). Non-specific antibody binding was blocked by incubation with a cocktail containing anti-FcγRIII/II mAb (BioLegend), mouse, hamster and rat serum. Subsequently, cells were stained with directly labeled anti-CD90.2, anti-CD44, anti-CD4, and anti-CD8a antibodies for 20 min at 4°C. After washing, cells were fixed and permeabilized over night with Cytofix/Cytoperm™ (BD Biosciences). Cells were washed with Perm/Wash buffer™ (BD Biosciences) and stained with directly labeled anti-IFNγ, anti-IL-10, anti-IL-17A, anti-IL-2, and anti-TNFα antibodies for 45 min at 4°C.

### Histology

Superior lobes of lungs from infected mice were fixed with 4% w/v PFA for 24 h and embedded in paraffin. Sections (4 μm) were rehydrated by running through xylenes, alcohols of decreasing concentrations and finally water. Sections were stained with hematoxylin and eosin (Merck) and/or carbol fuchsin (Merck) followed by decolorization with acid-alcohol to visualize mycobacteria in the lungs and analyzed with a BX41 light microscope and cellˆB software. Histological sections of infected lungs were scored in a blinded manner. Affected lung area was quantified in relation to whole lung area using cellˆB area measurement.

### RNA Isolation, cDNA-Synthesis, and Quantitative Real-Time PCR

Total RNA from lung tissue was extracted using TRIzol^®^ reagent (Invitrogen) and the Direct-zol™ RNA MiniPrep Kit (Zymo Research) as recommended by the manufacturer. For quantitative real-time PCR, 400 ng of total RNA were reverse transcribed using Maxima First Strand cDNA Synthesis Kit for RT-qPCR (Life Technologies) according to the manufacturer’s instruction at 25°C for 10 min, 55°C for 30 min, 85°C for 3 min. Real-time quantitative PCR reactions were performed using LightCycler^®^ 480 SYBR Green I Master (Roche). PCR amplifications were performed in duplicates in a total volume of 10 μl, containing 1 μl cDNA sample, 0.2 μl of primer pairs (10 μM), 5 μl SYBR green mix, and 3.8 μl RNase/DNase-free water. Data analysis was performed using the LightCycler^®^ 480 instrument. The PCR cycling conditions used were as follows: (I) Pre-incubation/denaturation: 95°C for 10 min; (II) Amplification: 45 cycles of 95°C for 10 s, 63°C for 10 s, 72°C for 8 s, and 72°C for 1 s (acquisition step). (III) Melt curve analysis: 95°C for 10 s, 65°C for 10 s, and gradual heating to 97°C with continuous fluorescence acquisition. (IV) A final cooling step at 37°C was included for handling of the samples, because the LightCycler has no cooling bloc. Analysis of the relative changes was performed using LightCycler480 Software 1.5.0 SP4 (Version 1.5.0.39, Roche). All quantifications were normalized to the level of HPRT gene expression (housekeeping gene). The following primers were used: HPRT forward TCCTCCTCAGACCGCTTTT and reverse CATAACCTGGTTCATCATCGC; IFNγ forward TCAAGTGGCATAGATGTGGAAGAA and reverse TGGCTCTGCAGGATTTTCATG; TNFα forward CCACCA CGCTCTTCTGTCTAC and reverse AGGGTCTGGGCCATA GAACT; IL-10 forward GGTTGCCAAGCCTTATCGGA and reverse ACCTGCTCCACTGCCTTGCT; IL-6 forward GAGGATACCACTCCCAACAGACC and reverse AAGTGCA TCATCGTTGTTCATACA; IL-12B forward CATCATCAAA CCAGACCCGCCCAA and reverse AACTTGAGGGAGAA GTAGGAATGG. Primers for iNOS were kindly provided by the group of Microbial Interface Biology.

### Multiplex Cytokine Assay

The concentrations of various cytokines in lung homogenates were determined by LEGENDplex™ (Mouse T helper cytokine panel and Mouse Inflammation panel, BioLegend) according to the manufacturer’s protocol.

### Nitric Oxide Assay

Nitric oxide was determined in lung homogenates as NO_2_^–^ using the Griess reagent (Sigma). Samples were mixed in equal volume with Griess reagent and incubated for 15 min. Optical density was measured at 560 nm with Tecan Sunrise Reader (Magellan).

### Isolation of CD11c^+^ Cells and *M. tuberculosis* Infection

C57BL/6 mice were infected with 1 × 10^5^ iRBC i.p. as described before. Fourteen days p.i. spleens from infected mice and naïve control mice, respectively, were harvested and digested in 100 μg/ml DNase I (Roche) and 50 μg/ml Liberase TL (Roche) in RPMI for 30 min and passed through a 100 μm pore size cell strainer. Remaining erythrocytes were lysed (155 mM NH_4_Cl, 10 mM KHCO_3_, 0.1 mM EDTA in H_2_O) and cells were resuspended in RPMI 1640 supplemented with 2 mM L-glutamine, 1% v/v Hepes, 50 μM β-mercaptoethanol and 10% v/v heat-inactivated fetal calf serum (complete RPMI 1640 medium). Dead cells were removed using the Dead Cell Removal Kit (Miltenyi) according to the manufacturer’s instruction. Afterwards, CD11c^+^ cells were magnetically labeled and isolated using CD11c MicroBeads (Miltenyi) as recommended by the manufacturer. 2 × 10^5^ CD11c^+^ cells were infected with *M. tuberculosis* in a MOI 1 directly from frozen stock. At indicated time points cells were lysed with 0.5% (v/v) Triton X-100 in PBS and tenfold serial dilutions of organ homogenates in sterile water/1% v/v Tween 80/1% w/v albumin were plated onto Middlebrook 7H11 agar plates.

### T Cell Proliferation Assay

CD11c^+^ cells isolated as described above were incubated with 100 μg/ml Ovalbumin (Sigma–Aldrich) over night or 1 μM OT-II peptide (peptide synthesis, Research Center Borstel) for 3 h. Spleens of transgenic OT-II mice, which contain CD4^+^ T cells specific for chicken ovalbumin 323–339, were harvested, passed through a 100 μm pore size cell strainer and erythrocytes were lysed (155 mM NH_4_Cl, 10 mM KHCO_3_, 0.1 mM EDTA in H_2_O). OT-II CD4^+^ T cells were isolated using CD4^+^ T cell isolation kit (Miltenyi; untouched) according to the manufacturer’s instruction. T cells were further labeled with CFSE (Invitrogen) and 1 × 10^5^ were co-incubated with 1 × 10^3^ CD11c^+^ cells for three days at 37°C.

### Statistical Analysis

Statistical analysis was performed by Mann–Whitney test or by Kruskal–Wallis test followed by Dunn’s Multiple Comparison test as described in the figure legends. Correlation between variables was determined by calculating Pearson’s coefficient using a 2-tailed analysis. *In vitro* CFU data were log transformed and analyzed by unpaired student’s *t*-test. All data were analyzed using GraphPad Prism 5 (GraphPad Software, Inc.).

## Results

### *P. yoelii* Infection Exacerbates Chronic *M. tuberculosis* Infection

**FIGURE 1:**
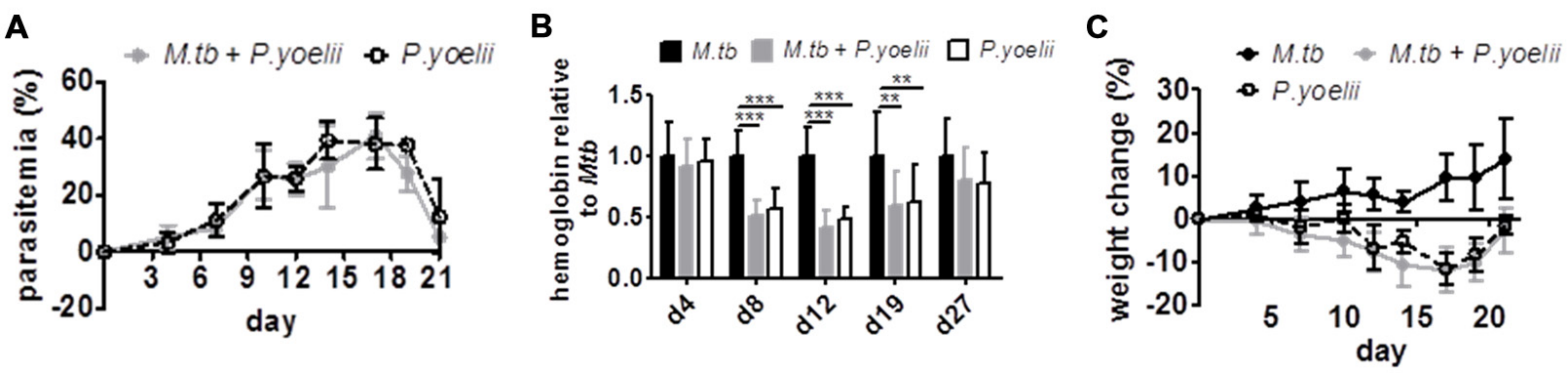
**The course of *P. yoelii* infection is unchanged.** C57BL/6 mice were infected via the aerosol route with *M. tuberculosis* H37Rv, and 30 days later with 1 × 10^5^ iRBCs i.p. **(A,C)** Parasitemia and weight were determined every 2–3 days starting 4 days after *P. yoelii* infection (groups of 7–8; one experiment representative out of three). **(B)** Starting 4 days after *P. yoelii* infection, hemoglobin levels were determined by collecting blood from tail vain, diluting in Drabkin’s Solution supplemented with Brij L23 Solution and measured at 540 nm (groups of 13–24). Statistical analysis was performed using the Kruskal–Wallis test with Dunn’s multiple comparison (data represent mean ± SD). ^∗∗^*p* < 0.01; ^∗∗∗^*p* < 0.001.

Due to its immunomodulatory properties we hypothesized that *P. yoelii* co-infection would interfere with immune control of chronic *M. tuberculosis* infection in C57BL/6 mice. Therefore, mice were infected via the aerosol route with a low dose of *M. tuberculosis* H37Rv. Thirty days later, when the immune response against *M. tuberculosis* was fully established and Tb in a chronic state, mice were infected with 1 × 10^5^ *P. yoelii* infected red blood cells (iRBCs) i.p. Parasitemia was monitored every 2–3 days on Giemsa stained thin blood smears starting 4 days after *P. yoelii* infection. Furthermore, anemia was assessed during acute *P. yoelii* infection by measuring hemoglobin levels in the blood. As expected, with rising parasitemia, hemoglobin levels significantly dropped in *P. yoelii* infected animals when compared to animals infected with *M. tuberculosis* (**Figures 1A,B**). However, we did not detect significant differences in parasitemia, anemia, and weight change between *P. yoelii* singly and co-infected animals (**Figures 1A–C**). Hence, the course of *P. yoelii* malaria does not change in mice chronically infected with *M. tuberculosis*.

**FIGURE 2:**
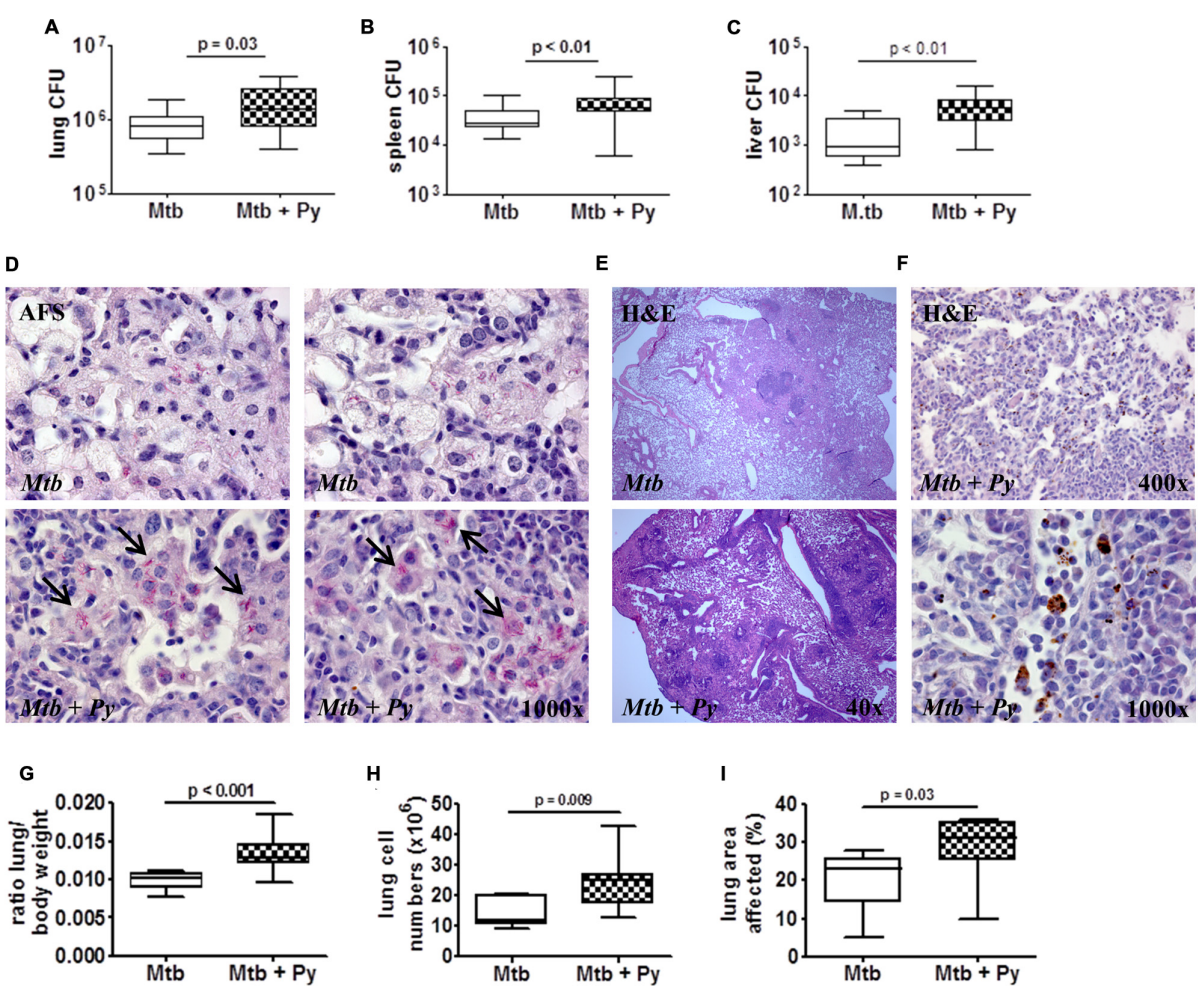
***P. yoelii* co-infection leads to increased lung pathology and *M. tuberculosis* burden.** C57BL/6 mice were infected via the aerosol route with *M. tuberculosis* H37Rv, and 30 days later with 1 × 10^5^ iRBCs i.p. Mice were sacrificed 21 days after *P. yoelii* infection for CFU determination in lung, spleen, and liver (**A–C**; lung and spleen groups of 16–23, liver groups of 9–15; data pooled from 2 to 3 independent experiments). Superior lung lobes were paraffin-embedded and 4 μm sections were acid fast and H&E stained to visualize *M. tuberculosis* **(D)** and hemozoin distribution (**F**; dark brown pigment) and to assess pulmonary inflammation **(E)**. Arrows indicate mycobacterial clusters. One or two representative images per group are shown (*n* = 7–8). **(G)** Lung to body weight ratio was determined in two independent experiments (*n* = 9–15). **(H)** Total lung cell numbers (*n* = 7–8). **(I)** Total area occupied by inflammatory lesions per lobe was quantified using cellˆB software (*n* = 7–8). Data are shown as box and whisker plots with the median and analyzed statistically using the Mann–Whitney test **(A–C,G–I)**.

Fifty-one days after *M. tuberculosis* infection, when *P. yoelii* infection was almost cleared, mice were sacrificed and lung, spleen, and liver were removed to analyze mycobacterial load. Compared to *M. tuberculosis* only infected mice, co-infected mice presented with a slight but statistically significant increase in *M. tuberculosis* burden in all three organs (**Figures 2A–C**). To further evaluate *M. tuberculosis* loads in the lung tissue, superior lung lobes were paraffin-embedded and sections were acid fast stained to visualize *M. tuberculosis*. Notably, while single bacteria were found across the *M. tuberculosis*-infected lungs, co-infected mice frequently harbored large clusters of mycobacteria (**Figures 2D**, bottom, arrows), indicating that CFU values were most likely underestimating actual *M. tuberculosis* numbers in co-infected lungs. In conclusion, these data suggest that malaria limits control of *M. tuberculosis.*

Mycobacterial infection leads to cellular infiltrations and formation of granulomatous lesions in the lung which are necessary to restrict and control the infection. However, excessive pathology also results in disease exacerbation (O’Garra et al., 2013). To investigate histopathological changes in single- and co-infected lungs, superior lung lobes were paraffin-embedded and 4 μm sections were H&E stained. Lungs of co-infected mice displayed increased pulmonary infiltration (**Figure 2E**) and increased total lung weight compared with lungs of mice infected with *M. tuberculosis* alone (**Figure 2G**). This was in line with a significant rise of absolute cell numbers in co-infected lungs compared to those of control animals (**Figure 2H**). In depth microscopic evaluation of lung sections revealed the deposition of hemozoin (**Figure 2F**), the malaria pigment which is produced by the parasite during digestion of red blood cell hemoglobin, in lungs of co-infected mice. Moreover, histopathological changes were more pronounced in co-infected lungs as reflected by more granulomatous lesions compared to *M. tuberculosis* infected lungs. Consequently, the total lung area affected was significantly increased upon *P. yoelii* co-infection compared to *M. tuberculosis* single infection (**Figure 2I**).

### *P. yoelii* Co-infection Augments Cytokine Responses in the Lung

**FIGURE 3:**
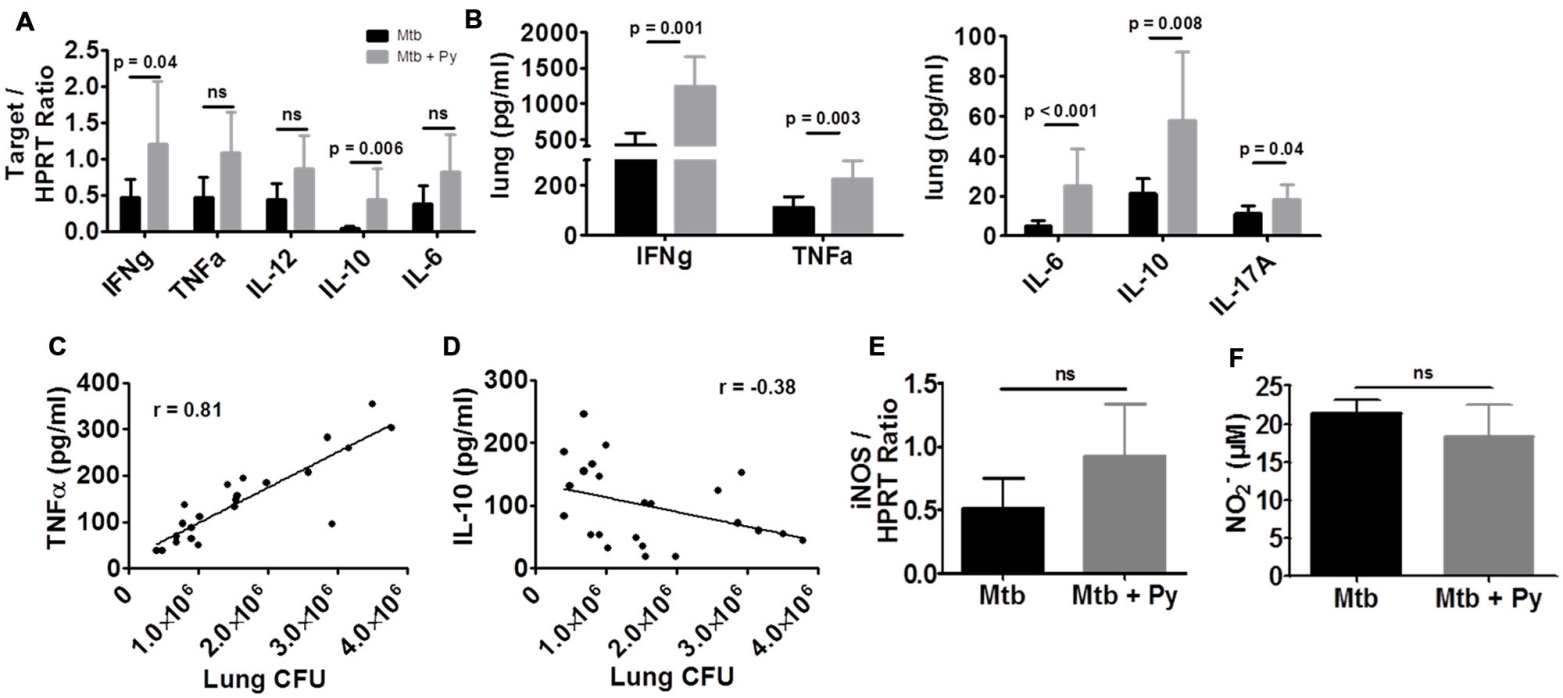
***P. yoelii* co-infection augments cytokine responses in the lung.** C57BL/6 mice were infected via the aerosol route with *M. tuberculosis* H37Rv, and 30 days later with 1 × 10^5^ iRBCs i.p. Lungs were collected 21 days after *P. yoelii* infection for analysis of cytokine **(A)** or iNOS **(E)** expression by quantitative RT-PCR (expression relative to housekeeping gene HPRT). **(B)** Cytokine protein levels were measured in lung lysates in *M. tuberculosis* and co-infected mice using LEGENDplex. **(F)** Nitric oxide production was determined by Griess reagent in lung lysates. Data represent one experiment representative for 2–3 independent ones and are shown as mean ± SD (*n* = 5–10; Mann–Whitney test). **(C)** TNFα and **(D)** IL-10 protein levels were correlated to lung CFU using Pearson’s correlation.

The balance of pro- and anti-inflammatory cytokines is necessary to restrict mycobacterial growth as well as to avoid immunopathology and maintain tissue function (O’Garra et al., 2013). To assess the effects of *P. yoelii* co-infection on different pro- and anti-inflammatory cytokines, their expression was determined on RNA and protein level (**Figures 3A,B**). Overall cytokine responses were elevated in co-infected compared to single infected mice. On mRNA level we found a significant increase in IFNγ and IL-10 expression (**Figure 3A**), whereas on protein level IFNγ, TNFα, IL-6, IL-10, and IL-17A were significantly increased compared to *M. tuberculosis* infected mice (**Figure 3B**). Correlation analysis revealed that increased TNFα levels positively correlated with increased *M. tuberculosis* CFU in lungs of co-infected mice (*r* = 0.81, Pearson’s correlation; **Figure 3C**). In contrast, elevated IL-10 levels did not correlate with increased CFU in co-infected animals (**Figure 3D**). IL-10 is known to exert anti-inflammatory functions including down-modulation of macrophage effector functions (Gazzinelli et al., 1992; Murray et al., 1997; Murray and Young, 1999). Increased IL-10 expression in co-infection mice thus prompted us to determine inducible nitric oxide synthase (iNOS) expression and NO levels in lung tissue. Neither the mRNA expression of iNOS nor the release of NO was decreased in lungs of co-infected mice suggesting that this host defense mechanism was not impaired during *P. yoelii* co-infection (**Figures 3E,F**).

### T Cell Response is Not Impaired in Co-infected Mice

**FIGURE 4:**
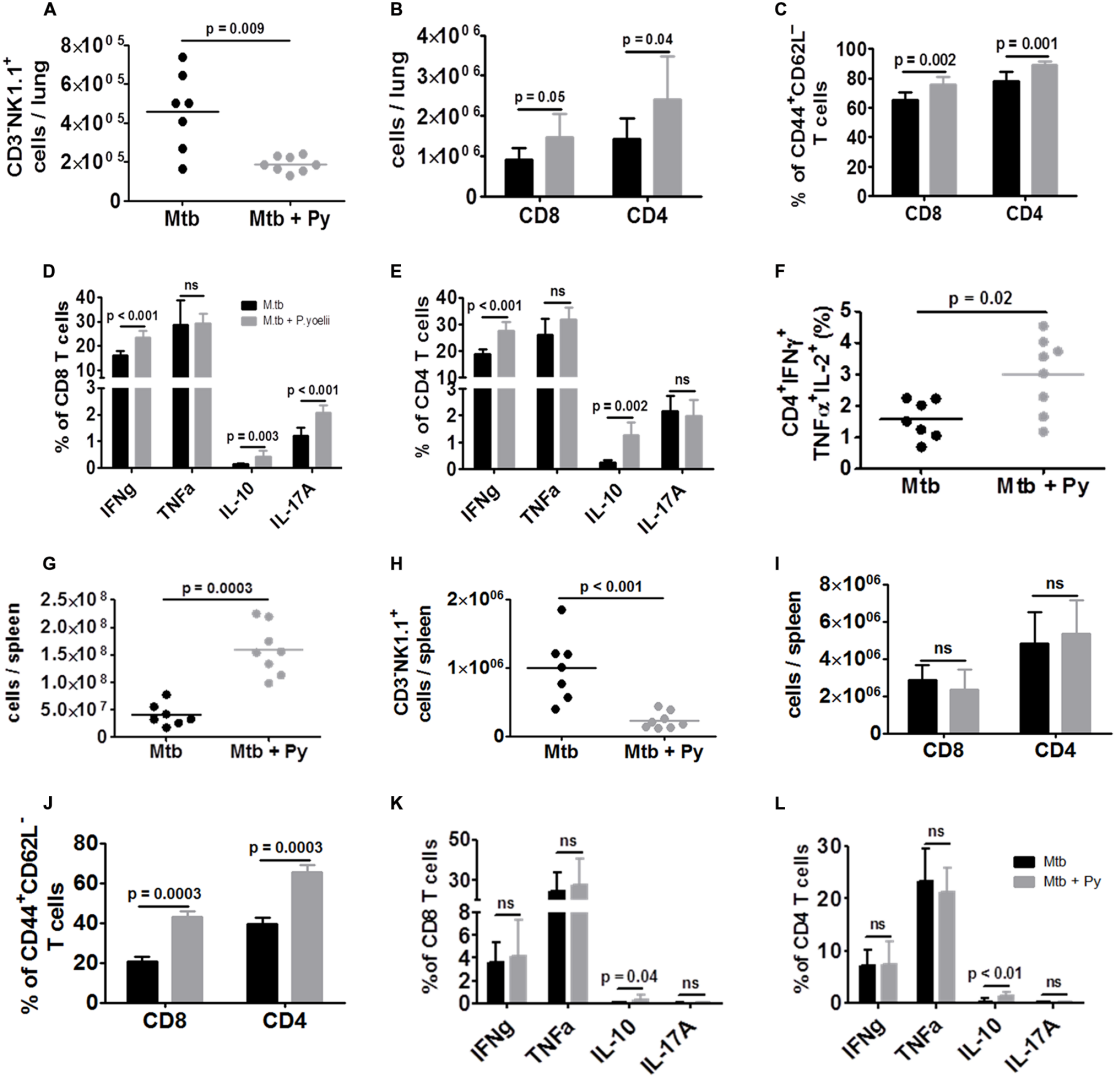
**T cell responses are not impaired in co-infected mice.** C57BL/6 mice were infected via the aerosol route with *M. tuberculosis* H37Rv, and 30 days later with 1 × 10^5^ iRBCs i.p. Lungs were collected 21 days after *P. yoelii* infection and single cell suspensions were analyzed for the presence and activation status of NK cells, CD4^+^ and CD8^+^ T cells by flow cytometry. **(A)** Lung cells were analyzed for CD3^–^NK1.1^+^ and **(B)** gated on CD90.2 to determine the total numbers of CD4^+^ and CD8^+^ T cells as well as **(C)** the proportion of effector T cells (CD44^+^CD62L^–^). **(D–F)** Lung cells were restimulated *ex vivo* with αCD3/αCD28 (5 μg, respectively) and analyzed by flow cytometry for the presence of IFNγ, TNFα, IL-10, IL-17A, and IFNγ/TNFα/IL-2 producing CD4^+^ and CD8^+^ T cells. **(G)** Total spleen cell numbers. **(H)** Spleen cells were analyzed for CD3^–^NK1.1^+^ and **(I)** gated on CD90.2 to determine total numbers of CD4^+^ and CD8^+^ T cells as well as **(J)** the proportion of effector T cells (CD44^+^CD62L^–^). **(K,L)** Spleen cells were restimulated *ex vivo* with αCD3/αCD28 (5 μg, respectively) and analyzed by flow cytometry for the presence of IFNγ, TNFα, IL-10, and IL-17A. Symbols and bars represent individual mice or means ± SD, respectively (*n* = 7–8; Mann–Whitney test). For full gating strategies, see Supplementary Figures S1A–C.

Histopathological alterations and elevated numbers of total lung cells together with elevated cytokine levels indicated increased immune cell recruitment and activity in the lungs of co-infected mice. Thus, we investigated the cell-mediated immune responses by flow cytometry. NK cells, defined as CD3^–^NK1.1^+^ cells, were significantly decreased in lungs of co-infected compared to *M. tuberculosis* infected mice (**Figure 4A**). In contrast, the numbers of both CD8^+^ and CD4^+^ T cells were significantly increased in the presence of *P. yoelii* (**Figure 4B**). Further analysis revealed significantly higher frequencies of CD4^+^ and CD8^+^ effector T cells (CD44^+^CD62L^–^) in co-infected lungs which produced significantly more cytokines upon *ex vivo* re-stimulation with αCD3/αCD28 (**Figures 4C–E**). Moreover, multifunctional CD4^+^ T cells, which have been associated with protection against Tb (Forbes et al., 2008) and secrete IFNγ, TNFα, and IL-2, were also significantly increased in the lungs of co-infected mice compared to *M. tuberculosis* mice (**Figure 4F**).

As the spleen plays a pivotal role in the development of the immune response against *Plasmodium* infection and in elimination of iRBC, we studied the cell-mediated immune response in this organ by flow cytometry. The overall number of splenocytes was significantly increased during co-infection (**Figure 4G**), reflecting splenomegaly associated with *P. yoelii* infection. As in the lung, NK cell numbers were significantly decreased in co-infected compared to *M. tuberculosis* infected mice (**Figure 4H**). There was no difference in the numbers of both splenic CD8^+^ and CD4^+^ T cells between co-infected and *M. tuberculosis* infected mice (**Figure 4I**). Although frequencies of effector CD8^+^ and CD4^+^ T cells (CD44^+^CD62L^–^) were significantly higher in co-infected spleens they did not produce more cytokines upon *ex vivo* re-stimulation with αCD3/αCD28 (**Figures 4J–L**) compared to splenic T cells from *M. tuberculosis* infected mice. Solely, the production of IL-10 was significantly increased.

In conclusion, while NK cell numbers were significantly decreased in lungs and spleens, T cell frequencies and function were increased in lungs when *P. yoelii* was concurrent with *M. tuberculosis*.

### CD11c^+^ Cells are Induced by *P. yoelii* Co-infection and Promote Survival of *M. tuberculosis In Vitro*

**FIGURE 5:**
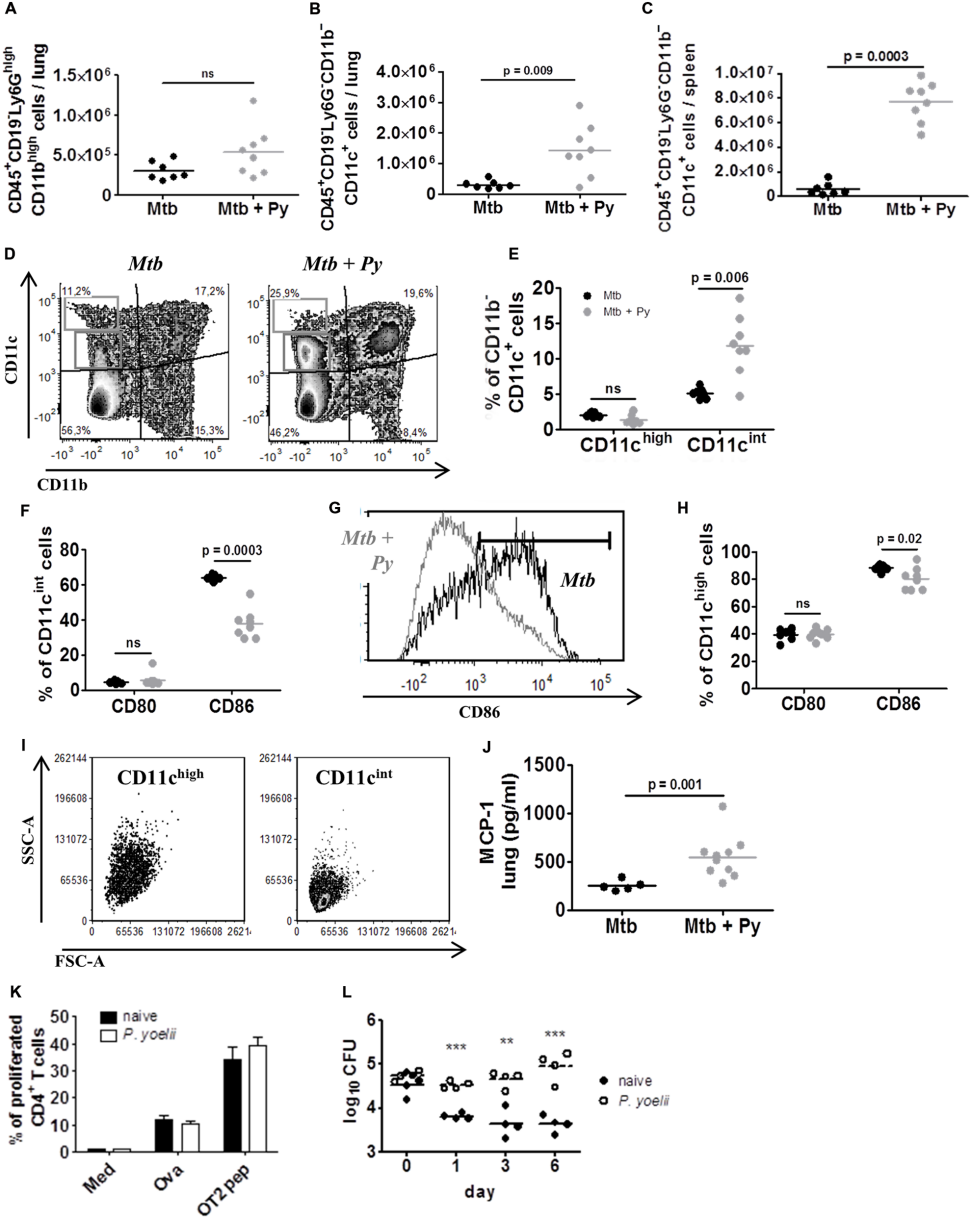
**CD11c^+^ cells are induced by *P. yoelii* co-infection and promote survival of *M. tuberculosis in vitro*.** C57BL/6 mice were infected via the aerosol route with *M. tuberculosis* H37Rv, and 30 days later with 1 × 10^5^ iRBCs i.p. Lungs and spleens were collected 21 days after *P. yoelii* infection and single cell suspensions were analyzed for the presence and activation status of Ly6G^+^ and CD11c^+^ cells by flow cytometry. **(A)** Lung cells were gated on CD45^+^ and further on CD19^–^ cells and analyzed for the presence of Ly6G^high^CD11b^high^ neutrophils. Symbols and bars represent individual mice and means, respectively (groups of 7–8). **(B)** Lung and **(C)** spleen cells were gated on CD45^+^ and further on CD19^–^ and Ly6G^–^ cells and analyzed for the presence of CD11b^+^ and CD11c^+^ cells. **(D–H)** Pulmonary CD11b^–^CD11c^+^ cells were further divided into CD11^cint^ and CD11c^high^ **(D,E)**. CD80 and CD86 expression was analyzed in both CD11b^–^CD11c^+^ populations **(F–H).** CD11c^int^ and CD11c^high^ populations were characterized in size (FSC-A) and granularity (SSC-A) **(I)**. Symbols and bars represent individual mice and means, respectively (*n* = 7–8; Mann–Whitney test). For full gating strategy, see Supplementary Figure S1D. **(J)** MCP-1 protein level was measured in lung lysates in *M. tuberculosis* and co-infected mice using LEGENDplex. **(K,L)** C57BL/6 mice were infected with *P. yoelii* and 14 days p.i., CD11c^+^ cells were isolated from spleens of infected and naïve control mice using magnetically labeled beads. Isolated CD11c^+^ cells were loaded with the model antigen Ova or Ova-derived peptides and co-cultured with CFSE-labeled transgenic OT-II T cells for 3 days. Loss of CFSE as indicator for T cell proliferation was measured by flow cytometry (**K**; performed in triplicates). In addition, isolated CD11c^+^ cells from naïve or malaria infected mice were infected with *M. tuberculosis* (MOI 1) and plated on 7H11 agar plates at indicated time points (**L**, one representative experiment out of three). Symbols and bars represent individual mice and means ± SD, respectively. Unpaired student’s *t*-test. ^∗∗^*p* < 0.01, ^∗∗∗^*p* < 0.001.

We next studied the influence of co-infection on the innate immune compartment of the lung via flow cytometry. We could observe a slight but not significant increase in CD45^+^CD19^–^Ly6G^high^CD11b^high^ cells (**Figure 5A**) indicating that neutrophils did not contribute to increased cellular infiltration in lungs from co-infected mice. In contrast, we found significantly more CD45^+^CD19^–^Ly6G^–^CD11b^–^CD11c^+^ cells in lungs from co-infected compared to *M. tuberculosis* infected mice (**Figure 5B**). This was even more pronounced in the spleen (**Figure 5C**). The pulmonary CD11b^–^CD11c^+^ compartment could be further divided into CD11c^high^ and CD11c^int^ cells (**Figure 5D**). While the CD11c^high^ population remained unchanged during co-infection, the frequency of CD11c^int^ cells significantly increased in the presence of *P. yoelii* (**Figure 5E**). Analysis of co-stimulatory molecules revealed reduced surface expression of CD86 on CD11c^int^ cells from co-infected as compared to *M. tuberculosis* infected mice (**Figures 5F,G**). Likewise, CD11c^high^ cells, although unchanged in numbers, exhibited a significant although less prominent reduction in the expression of CD86 (**Figure 5H**). When we evaluated both populations for their forward (FSC-A) and side scatter (SSC-A) pattern, CD11c^int^ cells were FSC-A^low^/SSC-A^low^ while CD11c^high^ cells were FSC-A^low^/SSC-A^high^, indicating increased granularity of CD11c^high^ cells compared with CD11c^int^ cells (**Figure 5I**). Alterations in leukocyte recruitment in co-infected mice prompted us to investigate chemokine protein levels in lung tissue. We found significantly elevated concentrations of monocyte chemoattractant protein-1 (MCP-1), one of the key chemokines that regulate migration and infiltration of monocytes/macrophages and dendritic cells (DCs; **Figure 5J**). Together, our data indicate that *P. yoelii* co-infection induces the overproduction of MCP-1 and the recruitment of CD11b^–^CD11c^int^ cells to the lungs of *M. tuberculosis* infected mice.

*Plasmodium* infection is known to modulate the function of phagocytic cells (Ocana-Morgner et al., 2003; Orengo et al., 2008; Hawkes et al., 2010), which is also indicated by the down-regulation of CD86 expression on CD11c^+^ cells from co-infected mice shown herein. To further analyze the function of malaria-induced CD11c^+^ cells, we investigated their ability to stimulate T cell proliferation *in vitro*. To do so, C57BL/6 mice were infected with *P. yoelii* and 14 days p.i. when parasitemia peaks (**Figure 1A**), CD11c^+^ DCs were isolated from spleens of infected and naïve control mice using magnetically labeled beads. Subsequently, isolated DCs were loaded with the model antigen ovalbumin (Ova) or Ova-derived peptides and co-cultured with CFSE-labeled transgenic OT-II T cells for 3 days. DCs from naïve and *P. yoelii* infected spleens were able to induce T cell proliferation equally well (**Figure 5K**), indicating that DCs from malaria-infected mice are fully functional antigen presenting cells. We next infected DCs from naïve or malaria infected mice with *M. tuberculosis* (MOI 1) and monitored intracellular survival over time. Noteworthy, while DCs from naïve animals were able to restrict *M. tuberculosis* survival CD11c^+^ DCs from *P. yoelii* infected mice supported *M. tuberculosis* survival and growth, resulting in a 1.5 log difference in CFU after 6 days (**Figure 5L**).

In conclusion, *P. yoelii* co-infection induces an increase in CD11c^+^ cells in lungs and spleens which support the growth of *M. tuberculosis in vitro*.

### *P. yoelii* Induced Exacerbation of Tb Disease is Transient

Exacerbated lung pathology together with elevated *M. tuberculosis* burden in lung, spleen, and liver in co-infected mice prompted us to analyze the long-term consequences of *P. yoelii* co-infection. To do so, mice were sacrificed 200 days after *M. tuberculosis* infection (170 days after *P. yoelii* infection). While regression of splenomegaly was observed in mice infected with *P. yoelii* alone spleens of co-infected mice were still enlarged. Likewise, spleens and livers of co-infected mice showed darker pigmentation resulting from the accumulation of hemozoin produced by the parasite during digestion of red blood cell hemoglobin while pigmentation was very much reduced in *P. yoelii* singly infected mice (data not shown). These observations indicate that the full resolution of malaria-related syndromes was delayed in *M. tuberculosis* infected mice.

**FIGURE 6:**
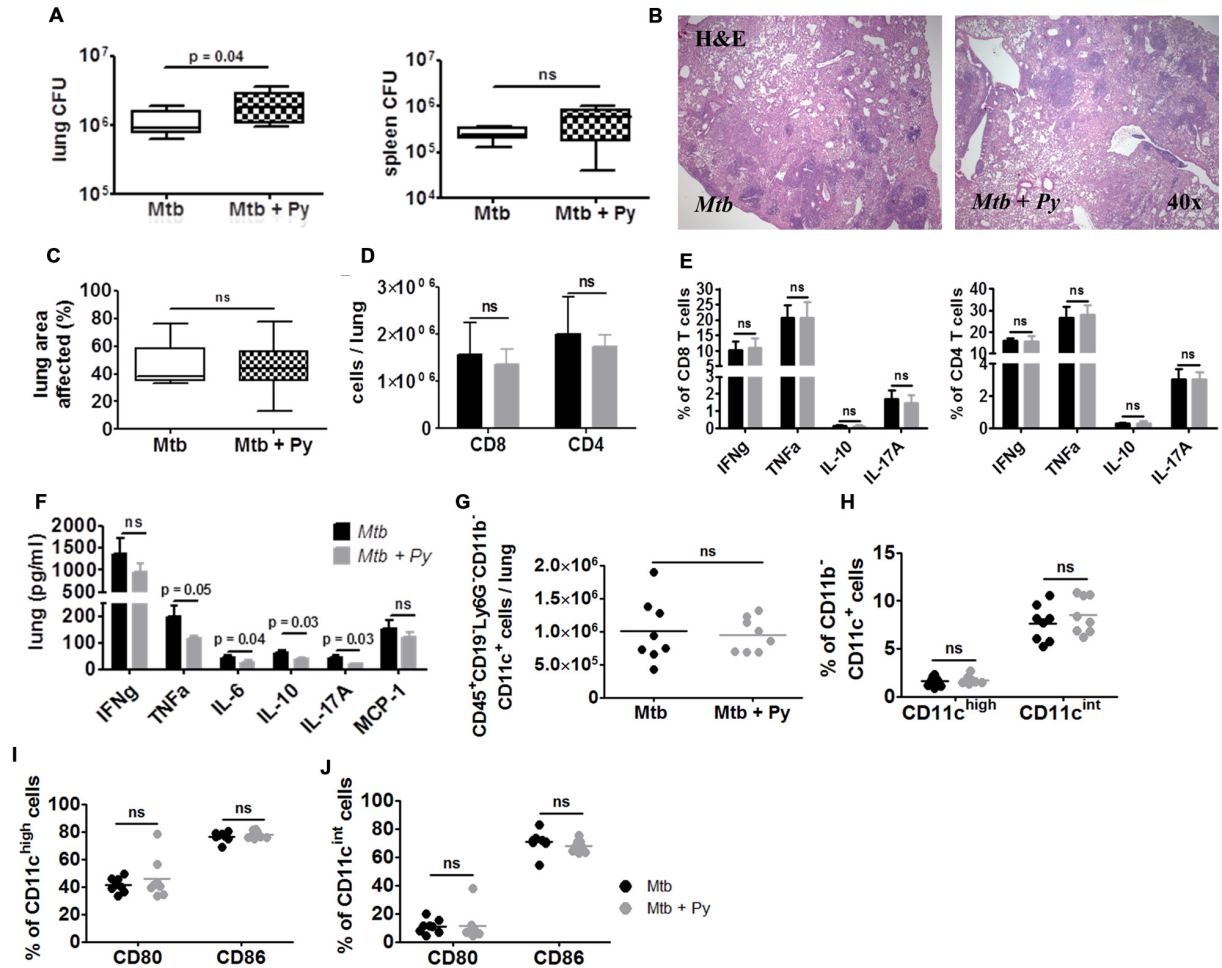
***P. yoelii* induced exacerbation of Tb disease is transient.** C57BL/6 mice were infected via the aerosol route with *M. tuberculosis* H37Rv, and 30 days later with 1 × 10^5^ iRBCs i.p. Lungs and spleens were collected 170 days after *P. yoelii* infection. **(A)** Lung and spleen CFU was determined. Data are shown as box and whisker plots with the median and analyzed statistically using Mann–Whitney test (*n* = 8). **(B,C)** Superior lung lobes were paraffin-embedded, and 4 μm sections were H&E-stained (one representative image per group is shown). Pulmonary inflammation was quantified using cellˆB software (*n* = 8; box and whisker plots with the median; Mann–Whitney test). Lung single cell suspensions were analyzed for the presence **(D)** and function **(E)** of CD4^+^ and CD8^+^ T cells by flow cytometry (restimulated *ex vivo* with αCD3/αCD28). **(F)** Cytokine protein levels were measured in lung lysates in *M. tuberculosis* and co-infected mice using LEGENDplex. **(G–J)** Lung cells were gated on CD45^+^ and further on CD19^–^ and Ly6G^–^ cells and analyzed for the presence of CD11b^+^ and CD11c^+^ cells. CD11b^–^CD11c^+^ cells were further divided into CD11c^int^ and CD11c^high^ **(H)**. CD80 and CD86 expression was analyzed in both CD11b^–^CD11c^+^ populations **(I,J)**. For full gating strategy, see Supplementary Figures S1A,B,D. Data are shown as means ± SD and were analyzed using the Mann–Whitney test (*n* = 8).

Determination of mycobacterial CFU revealed that co-infected mice still presented with a statistically significant but slight increase in *M. tuberculosis* burden in the lung, but not in the spleen (**Figure 6A**). Microscopic evaluation of lung sections revealed no differences in histopathology between *M. tuberculosis* singly and co-infected mice (**Figure 6B**). Inflammation had progressed over time, affecting approximately half of the lung area of mice from both groups (**Figure 6C**). In line with the only slightly increased *M. tuberculosis* burden and comparable histopathological changes in the lungs, mice from both groups had survived until this time-point of analysis without any clinical signs of disease.

In good agreement with histological observations, we could no longer observe differences in inflammatory immune responses in the lungs between both groups. Comparable numbers of CD8^+^ and CD4^+^ T cells were recovered from *M. tuberculosis* or co-infected lungs, which produced comparable amounts of cytokines upon *ex vivo* re-stimulation with αCD3/αCD28 (**Figures 6D,E**). Accordingly, cytokine levels in lung homogenates were no longer increased in co-infected mice. In contrast, levels of TNFα, IL-6, IL-10, and IL-17A were significantly decreased in co-infected lungs (**Figure 6F**). Likewise, numbers of CD45^+^CD19^–^Ly6G^–^CD11b^–^CD11c^+^ cells were no longer elevated in lungs from co-infected compared to *M. tuberculosis*-infected mice (**Figure 6G**). The same was true for both CD11c^high^ and CD11c^int^ populations (**Figure 6H**). Moreover, reduced expression of CD86 on both CD11c^high^ and CD11c^int^ cells as observed 21 days after co-infection was no longer apparent at this late time-point (**Figures 6I,J**).

In conclusion, while the resolution of splenomegaly and clearance of hemozoin was delayed in co-infected mice, one episode of *P. yoelii* co-infection had no long-term consequences on disease progression and survival of *M. tuberculosis* infected mice.

## Discussion

Malaria and Tb are co-endemic in many regions in the world, however, compared to other co-infections like HIV/Tb or helminth/Tb, it has been given less attention both in clinical and immunological studies. Due to the lack of sufficient human data, the impact of malaria on Tb and *vice versa* is difficult to estimate but co-infections are likely to occur very frequently (Bates et al., 2015).

In this study, we demonstrate that co-infection with the self-resolving parasite *P. yoelii* can transiently exacerbate Tb disease severity although the effect on *M. tuberculosis* control was minimal as reflected by a moderate but nevertheless statistically significant increase in CFU recovered from lung, spleen, and liver of co-infected mice. It should, however, be noted that acid fast staining of tissue sections revealed mycobacterial aggregates in co-infected but rarely in singly infected lungs. Such aggregates were most likely incompletely resolved during organ homogenization and plating, indicating that CFU values were probably underestimating actual *M. tuberculosis* numbers in co-infected lungs (Lewin et al., 2003).

*P. yoelii* infection during chronic Tb mediated increased recruitment of immune cells to the lungs which coincided with enhanced production of pro- and anti-inflammatory cytokines. T cell responses were not impaired by co-infection but augmented. Upon *ex vivo* re-stimulation, CD4^+^ and CD8^+^ T cells from co-infected lungs produced more IFNγ and IL-10. IL-10 is a negative regulator of Th1 responses and of central importance in immunity to malaria, where it ameliorates immunopathology at the expense of parasite elimination (Couper et al., 2008a,b; Freitas do Rosario et al., 2012). Likewise, IL-10 antagonizes pro-inflammatory responses essential for protective immunity to *M. tuberculosis* (Murray et al., 1997; Murray and Young, 1999; Boussiotis et al., 2000; Schreiber et al., 2009; Redford et al., 2010) including IFNγ induced production of reactive nitrogen intermediates (RNI; Gazzinelli et al., 1992) which mediate the killing of *M. tuberculosis* (Chan et al., 1992; MacMicking et al., 1997; Herbst et al., 2011). However, iNOS expression and NO levels in lungs were not altered by *P. yoelii* co-infection, which might be one reason why *M. tuberculosis* control was only slightly impaired in co-infected mice. The importance of iNOS goes beyond direct killing of *M. tuberculosis* by RNI. During persistent infection, it is indispensable for modulating destructive inflammatory responses and its absence results in increased neutrophil recruitment and tissue necrosis at the site of *M. tuberculosis* infection (Chan et al., 1995; Cooper et al., 2000; Beisiegel et al., 2009; Mishra et al., 2013). In line with unchanged iNOS expression and despite an increase in IL-17A protein, which is known to induce neutrophil recruitment, we did not observe more neutrophils in co-infected lungs. Our data thus indicate that exacerbated immunopathology in the presence of *P. yoelii* was not neutrophil driven. The significant increase in IFNγ in co-infected lungs most likely contributes to the inhibition of pathogenic neutrophil accumulation (Nandi and Behar, 2011). IFNγ not only regulates the production of IL-17-induced chemokines, such as KC or MIP-2 but it suppresses expression of E- and P-selectin on endothelial cells which are important for neutrophil trafficking into inflamed tissue (Melrose et al., 1998; Desvignes and Ernst, 2009). Moreover, IFNγ can act on neutrophils directly by accelerating neutrophil death *in vitro* (Nandi and Behar, 2011). The source for IFNγ were most likely T cells, since NK cells, which contribute to early IFNγ production during *M. tuberculosis* infection (Korbel et al., 2008), were significantly decreased in numbers during co-infection. Depletion of NK cells prior to and during infection has no impact on control of mycobacterial growth (Junqueira-Kipnis et al., 2003). Only when T cells are lacking, NK cells can provide protection against *M. tuberculosis* infection to a certain extent and are crucial for the limitation of pathology (Feng et al., 2006). Because IFNγ levels were increased and T cell responses not impaired during *P. yoelii* co-infection, we reasoned that the reduction in NK cell numbers had no consequences on disease outcome.

Resolution of acute infections such as malaria relies on the stimulation of myelopoiesis in order to meet the need for an efficient innate immune response (Belyaev et al., 2013). During blood-stage malaria infection, innate cells such as monocytes, macrophages, and DCs are required in high numbers for the removal of parasitized red blood cells and T cell priming which takes place in the spleen (Langhorne et al., 2004, 2008; Voisine et al., 2010). Of these innate cells, CD11c^+^ DCs are involved in both the priming of T cells and the control of parasitemia (deWalick et al., 2007; Wykes et al., 2007; Voisine et al., 2010). Consequently, it was reported that numbers of CD11c^+^ DCs in the spleen rise considerably during infection with *P. yoelii* and other rodent malaria parasites (Langhorne et al., 2004; Wykes et al., 2007; Voisine et al., 2010). We found increased numbers of CD11b^–^CD11c^+^ cells in lungs and spleens of co-infected mice compared to mice infected with *M. tuberculosis* alone. While most CD11c^+^ cells represent conventional DCs (cDCs) in the spleen, the classification is more complicated in the lungs were alveolar macrophages also express CD11c (Lancelin and Guerrero-Plata, 2011; Kopf et al., 2015). Of the pulmonary CD11b^–^CD11c^+^ cells, only CD11c^int^ cells were significantly increased during co-infection while numbers of CD11c^high^ cells remained unchanged. According to their FSC–SSC pattern, we reasoned these cells are of DC-like nature as reflected by low granularity compared to the CD11c^hi^ population which most likely represents alveolar macrophages. Both CD11c^high^ and CD11^int^ cells displayed a significant reduction in CD86 surface expression in co-infected compared to those from *M. tuberculosis* infected mice, indicating that *P. yoelii* co-infection interfered with cell activation. This has been described before (Ocana-Morgner et al., 2003; Urban and Todryk, 2006; Orengo et al., 2008). Regardless, T cell responses were not impaired in co-infected animals. *M. tuberculosis* specific immune responses are primed by DCs in the lung-draining lymph node in the first 2 weeks of *M. tuberculosis* infection (Wolf et al., 2008). Thus, by the time of *P. yoelii* co-infection (30 days after *M. tuberculosis* infection), *M. tuberculosis* specific immune responses were already established.

Beside their role in T cell priming, DCs along with other phagocytes serve as host cell for *M. tuberculosis* in lungs of infected mice (Wolf et al., 2007). While activated macrophages are able to kill mycobacteria, DCs fail to eliminate them but rather promote their survival (Bodnar et al., 2001). In fact, when DC numbers were increased by treatment with polyethylene glycol-conjugated GM-CSF or Flt3-L in mice infected with *M. tuberculosis* the overall control of infection was impaired and mice had greater bacterial burden and mortality than controls (Alaniz et al., 2004). In the same study, the control of *Listeria* was also impaired by the induction of DCs which were shown to harbor viable bacteria. Likewise, *Salmonella* preferentially infect DCs which are unable to kill them (Marriott et al., 1999). The fact that DCs are less efficient killers than macrophages and more resistant to cytotoxic T cell lysis (Medema et al., 2001) makes them an attractive cellular niche for intracellular bacteria such as *M. tuberculosis*. Importantly, DCs from *P. yoelii* infected mice were much more permissive to *M. tuberculosis* survival and replication *in vitro* than DCs from naïve mice. These results suggest that *P. yoelii* induced DCs provide an environment in which intracellular *M. tuberculosis* thrive. The induction of an *M. tuberculosis*-permissive monocyte population has been recently reported in a study investigating the consequences of intranasal Poly-IC treatment of *M. tuberculosis* infected mice (Antonelli et al., 2010). Poly-IC, a synthetic analog of dsRNA, is a potent inducer of type I IFN responses and currently used in clinical trials due to its efficacy in viral infections and malignancies (Borden et al., 2007). Poly-IC treatment triggered the IFN-dependent pulmonary recruitment of a CD11b^+^F4/80^+^Gr1^int^ population that displayed enhanced mycobacterial levels. The authors suggest that Poly-IC treatment can detrimentally affect the outcome of *M. tuberculosis* infection by promoting the accumulation of a permissive myeloid population in the lung.

In addition to promoting *M. tuberculosis* survival, infected DCs can shape tissue pathology. Recently *M. tuberculosis* infected inflammatory DCs were shown to spread granulomatous inflammation in infected tissue. CD11c^+^ DCs left mycobacterial granulomas with bacteria and formed contact with *M. tuberculosis* specific T cells, thereby inducing new multi-focal lesions in the lungs (Harding et al., 2015). In our model, *P. yoelii* co-infection induced the recruitment of CD11c^+^ cells to *M. tuberculosis* infected lungs and the formation of more granulomatous lesions as compared to animals infected with *M. tuberculosis* alone. Thus, increased numbers of CD11c^+^ cells in co-infected lungs could promote dissemination of mycobacteria across the lungs and the formation of new lesions, thereby contributing to the exacerbated tissue pathology observed herein.

We believe that in our co-infection model, the stimulation of myelopoiesis together with the enhanced recruitment of myeloid progenitors from the bone marrow in the course of *P. yoelii* co-infection results in the enhanced recruitment of immune cells to the site of *M. tuberculosis* infection in the lung, most likely attracted by chemokines which are produced in response to *M. tuberculosis* infection. It has been shown that systemic IFNγ responses triggered the secretion of CCL2 (MCP-1) and CCL7 which led to the egress of early myeloid progenitors from the bone marrow during malaria infection (Belyaev et al., 2013). Significantly elevated levels of IFNγ and MCP-1 in lungs of co-infected mice could be responsible for enhanced pulmonary recruitment of myeloid cells. In addition, TNFα directly effects immune cell recruitment by upregulation of endothelial adhesion molecules (Zhou et al., 2007) and induction of chemokine production which further recruit leukocytes to the site of infection (Roach et al., 2002; Algood et al., 2005).

The deposition of hemozoin, the malaria pigment which is produced by the parasite during digestion of red blood cell hemoglobin, might also augment inflammatory responses in the lung of co-infected mice (Parroche et al., 2007; Deroost et al., 2013). During malaria infection, circulating and resident phagocytes take up and accumulate hemozoin, which is released into the circulation during erythrocyte lysis. This leads to accumulation of malaria pigment in different organs with potential immune modulating consequences. Moreover, some parasites including *P. falciparum* and the rodent strains *P. berghei* and *P. chabaudi* are known to sequester in host tissue with the lung being a major site of parasite sequestration while spleen and liver function in digestion of infected erythrocytes. Infection with the lethal strain *P. berghei* NK65 (*Pb*NK65) induces severe lung pathology and was described as a model for malaria-associated acute respiratory distress syndrome in mice (MA-ARDS; Van den Steen et al., 2010). Recently, an association has been found between increased levels of hemozoin in pulmonary tissue of *Pb*NK65 infected mice and MA-ARDS (Deroost et al., 2013). This severe lung pathology could be one reason why in *M. tuberculosis* infected mice, the consequences of *Pb*NK65 co-infection are much more severe as compared to *P. yoelii* co-infection (Mueller et al., 2012).

While we observed alterations in immune cell recruitment and immunopathology in *M. tuberculosis* infected mice shortly after acute *P. yoelii* co-infection, differences in immunopathology and cellular immune responses between *M. tuberculosis* and co-infected mice were no longer apparent 150 days later. This indicates that *P. yoelii* co-infection had only a transient effect on Tb disease severity, which is supported by the fact that mice from both groups did not show clinical signs of disease or premature death throughout the entire observation period. The reason for this is most likely the transient nature of the *P. yoelii* infection. Parasitemia is resolved within 3–4 weeks and consequently, *P. yoelii* induced immune responses decline over time. Moreover, hemozoin, which probably contributes to increased inflammation in the co-infected lungs, is known to be redistributed to liver and spleen over time (Levesque et al., 1999; Frita et al., 2012; Deroost et al., 2013).

Mice in our model only experienced one episode of *Plasmodium* infection. In contrast, people living in malaria-endemic settings are constantly reinfected with malaria parasites. Hence, these people potentially suffer from continuous immune modulation, which might increase their susceptibility to Tb. Studying malaria reinfections in our mouse model is complicated by the fact that C57BL/6 mice after recovering from parasitemia, become resistant to reinfection with *P. yoelii* (Lucas et al., 1993). This does not reflect the situation in humans where immunity to malaria develops relatively slowly and sterile immunity is probably never achieved (Langhorne et al., 2008). However, the fact that one single episode of *P. yoelii* co-infection is able to modulate immune responses and inflammation in the lungs of *M. tuberculosis* infected mice indicates that constant exposure to malaria could be a risk factor for Tb patients.

Importantly, our observations indicate that the full resolution of malaria-related syndromes was delayed in *M. tuberculosis* infected mice. The fact that co-infected mice, although able to clear parasitemia equally well, showed a delay in the resolution of splenomegaly and clearance of hemozoin from spleen and liver indicates that immune cell function in the spleen is modulated long-term. Since the spleen is the central organ for immunity in malaria, the question arises as to whether immunity to *P. yoelii* reinfection is established and/or maintained in *M. tuberculosis* infected mice. This important question will be addressed in our lab in future studies. These studies shall also reveal if recurrent *P. yoelii* infections will cause permanent immune modulation and ultimate loss of control of chronic *M. tuberculosis* infection.

In conclusion, one episode of *P. yoelii* co-infection transiently exacerbated Tb disease severity but had no long-term consequences on disease progression and survival of *M. tuberculosis* infected mice.

## Author Contributions

Conceived and designed the experiments: BS, JB, JBe, and TJ. Performed the experiments: JB, LE, and BS. Analyzed the data: JB and JBe. Contributed reagents/materials/analysis tools: BS, TJ, and JBe. Wrote the paper: BS and JB.

## Conflict of Interest Statement

The authors declare that the research was conducted in the absence of any commercial or financial relationships that could be construed as a potential conflict of interest.
