# Supplementary material for: One Episode of Self-Resolving Plasmodium yoelii Infection Transiently Exacerbates Chronic Mycobacterium tuberculosis Infection
Source: Front Microbiol. 2016 Feb 15;7:152. doi: 10.3389/fmicb.2016.00152 (PMC4753732; doi:10.3389/fmicb.2016.00152)
Supplement: Supplementary file 1 [file Image_1.PDF]

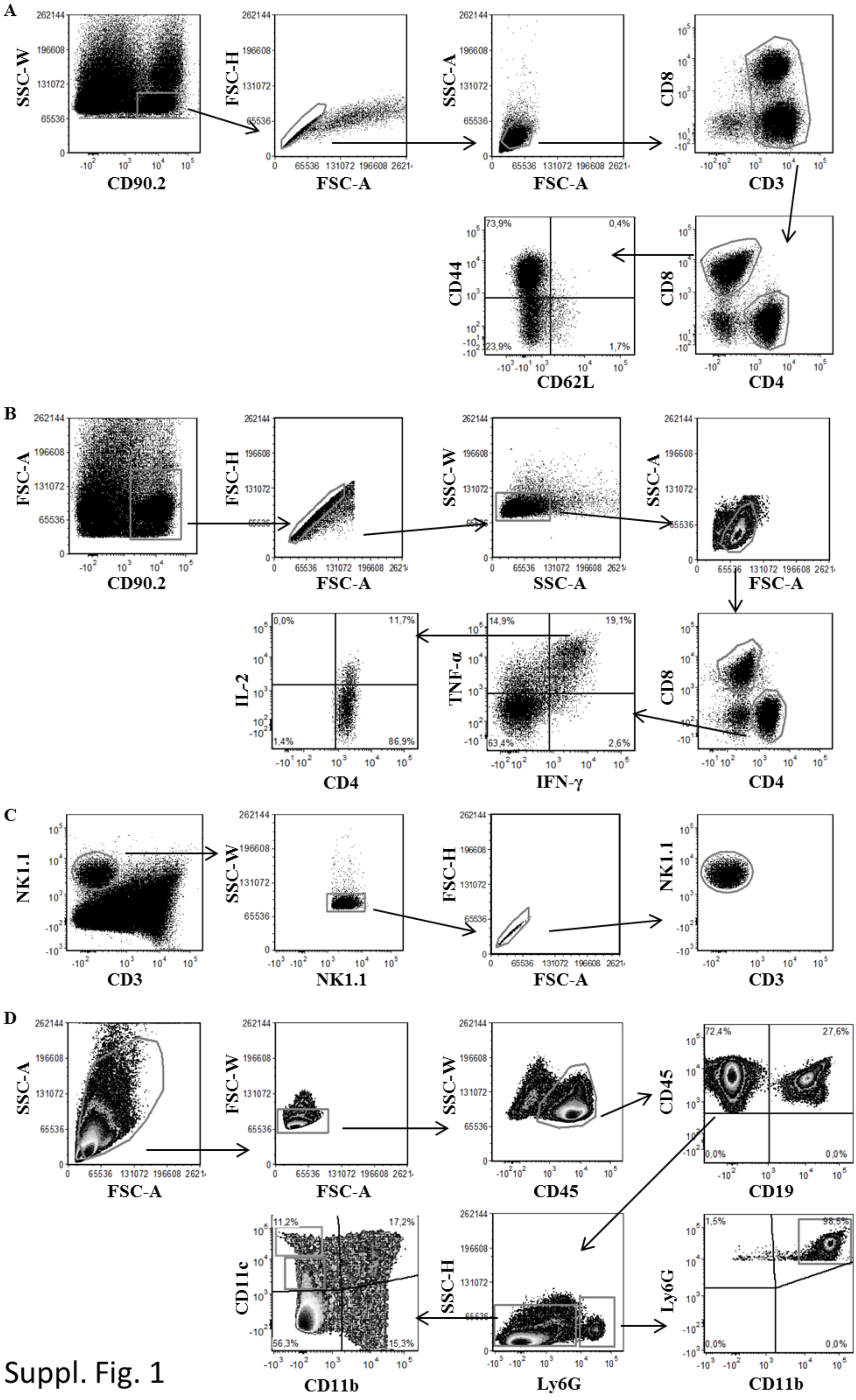
A
SSC-W
CD90.2
FSC-H
FSC-A
SSC-A
CD8
CD3
CD44
CD62L
73,9%
0,4%
23,9%
1,7%
CD4
B
FSC-A
CD90.2
FSC-H
SSC-W
SSC-A
IL-2
CD4
0,0%
11,7%
1,4%
86,9%
TNF-α
IFN-γ
14,9%
19,1%
63,4%
2,6%
CD8
C
NK1.1
CD3
SSC-W
FSC-H
D
SSC-A
FSC-W
SSC-W
CD45
CD19
72,4%
27,6%
0,0%
0,0%
CD11c
CD11b
11,2%
17,2%
56,3%
15,3%
SSC-H
Ly6G
1,5%
98,5%
0,0%
0,0%

Suppl. Fig. 1

Supplementary Figure 1: Gating strategies. A) Gating strategy for $CD8^+$ and $CD4^+$ T cells and effector T cells ($CD44^+CD62L^-$) shown in Figure 4B, C, I, J and 6D: Cells were stained for CD90.2, CD3, CD4, CD8, CD44 and CD62L. CD90.2 versus width of SSC was used to determine an accurate $CD90.2^+$ cell population and to exclude doublets. Further doublet and dead cell exclusion was performed by gating the $CD90.2^+$ population in FSC-A versus FSC-H and FSC-A versus SSC-A. CD3 versus CD8 was used to determine an accurate T cell population. CD4 or CD8 cells were gated and single and co-expression of CD62L and CD44 was determined to distinguish between specific cell phenotypes. B) Gating strategy for analysis of T cell cytokine production shown in Figure 4D-F, K, L and 6E: Cells were stained for CD90.2, CD4, CD8, IFNγ, TNFα, IL-10, IL-17A and IL-2. CD90.2 versus width of FSC was used to define a $CD90.2^+$ cell population which was subjected to three different gatings for doublet exclusion. Gating CD4 versus CD8 was used to determine accurate $CD8^+$ and $CD4^+$ cell populations, respectively, which were further assessed for their production of cytokines (shown here: IFNγ and TNFα; IL-10 and IL-17A production was assessed accordingly). $CD4^+$ T cells producing IFNγ and TNFα were analyzed for their property to produce IL-2. Where representative scatter plots show CD4 gating, $CD8^+$ cells were assessed accordingly. C) Gating strategy for NK cells shown in Figure 4A and H: Cells were stained for CD3 and NK1.1. CD3 versus NK1.1 was used to determine a population of NK cells which was further determined more precisely by two different gatings of doublet exclusion. Cells were finally gated on CD3 versus NK1.1 to obtain an accurate population. D) Gating strategy for $Ly6G^{high}CD11b^{high}$ and $CD11c^+$ cells shown in Figure 5 A-I and 6 H-J: Cells were stained for CD45, CD19, Ly6G, CD11b, CD11c, CD80 and CD86. FSC-A versus SSC-A, FSC-A versus FSC-W and finally CD45 versus SSC-A were used to determine an accurate $CD45^+$ cell population including doublet exclusion. CD19 versus CD45 was used to exclude B cells. $CD45^+CD19^-$ cells were further divided in $Ly6G^+$ and $Ly6G^-$ cells. CD11b versus Ly6G was used to determine an accurate $Ly6G^{high}CD11b^{high}$ population. CD11b versus CD11c was used to determine $CD11b^-CD11c^+$ cells which were further divided in $CD11c^{high}$ and $CD11c^{int}$ and analyzed for co-stimulatory molecule expression.
